# The calcium pump plasma membrane Ca^2+^-ATPase 2 (PMCA2) regulates breast cancer cell proliferation and sensitivity to doxorubicin

**DOI:** 10.1038/srep25505

**Published:** 2016-05-05

**Authors:** Amelia A. Peters, Michael J. G. Milevskiy, Wei C. Lee, Merril C. Curry, Chanel E. Smart, Jodi M. Saunus, Lynne Reid, Leonard da Silva, Daneth L. Marcial, Eloise Dray, Melissa A. Brown, Sunil R. Lakhani, Sarah J. Roberts-Thomson, Gregory R. Monteith

**Affiliations:** 1 School of Pharmacy, The University of Queensland, Brisbane, Queensland, 4072, Australia; 2 School of Chemistry and Molecular Biosciences, The University of Queensland, Brisbane, Queensland, 4072, Australia; 3 UQ Centre for Clinical Research, The University of Queensland, Herston, Queensland, 4006, Australia; 4 School of Medicine, University of Queensland, Brisbane, Queensland, 4072, Australia; 5 QIMR Berghofer Medical Research Institute, Cancer Genetics, Herston, Queensland, 4006, Australia; 6 School of Biomedical Sciences, Queensland University of Technology, Brisbane, Queensland, 4102, Australia; 7 Pathology Queensland, The Royal Brisbane & Women’s Hospital, Brisbane, Queensland, 4006, Australia; 8 Mater Research Institute, The University of Queensland, Brisbane, Queensland, 4072, Australia

## Abstract

Regulation of Ca^2+^ transport is vital in physiological processes, including lactation, proliferation and apoptosis. The plasmalemmal Ca^2+^ pump isoform 2 (PMCA2) a calcium ion efflux pump, was the first protein identified to be crucial in the transport of Ca^2+^ ions into milk during lactation in mice. In these studies we show that PMCA2 is also expressed in human epithelia undergoing lactational remodeling and also report strong PMCA2 staining on apical membranes of luminal epithelia in approximately 9% of human breast cancers we assessed. Membrane protein expression was not significantly associated with grade or hormone receptor status. However, PMCA2 mRNA levels were enriched in Basal breast cancers where it was positively correlated with survival. Silencing of *PMCA2* reduced MDA-MB-231 breast cancer cell proliferation, whereas silencing of the related isoforms *PMCA1* and *PMCA4* had no effect. *PMCA2* silencing also sensitized MDA-MB-231 cells to the cytotoxic agent doxorubicin. Targeting PMCA2 alone or in combination with cytotoxic therapy may be worthy of investigation as a therapeutic strategy in breast cancer. PMCA2 mRNA levels are also a potential tool in identifying poor responders to therapy in women with Basal breast cancer.

The enrichment of milk with calcium is vital to neonatal and infant development. The process by which calcium ions are transferred from the maternal blood supply into milk is highly coordinated, and involves specific calcium-permeable ion channels, and calcium pumps of both the secretory pathway and plasma membrane^1^,^2^,^3^,^4^,^5^,^6^. Recent studies have associated many of these specific calcium channels and pumps in processes important in breast cancer progression. Calcium signaling is a key regulator of many processes important in tumor progression including cellular proliferation, sensitivity to death stimuli, migration and invasion^7^. Indeed, specific calcium channels and pumps are identified as potential therapeutic targets in a number of cancer types including those of the prostate and breast^8^,^9^.

Expression of the canonical store-operated Ca^2+^ channel Orai1^10^ is increased during lactation in mice^1^. *In vitro* models^11^,^12^ and more recently Orai1-null mice studies^3^, suggest that Orai1 plays an important role in the basolateral influx of Ca^2+^ across mammary epithelial cells during lactation. Orai1 is also a potential drug target for some breast cancers on the basis of overexpression in some breast cancer cell lines^1^ and the ability of Orai1 silencing to reduce proliferation^1^,^13^, migration and invasion^14^ of breast cancer cells. Similarly, the secretory pathway Ca^2+^-ATPase isoform 2 (SPCA2) is associated with increased expression during lactation and specific breast cancer subtypes^2^,^13^. Silencing of SPCA2 reduces the proliferation of MCF-7 breast cancer cells *in vitro* and *in vivo*^13^.

The first specific protein identified as crucial in the process of Ca^2+^ ion transport during lactation was the plasmalemmal Ca^2+^ pump isoform 2 (PMCA2). In contrast to its related isoforms PMCA1 and PMCA4, PMCA2 has a restricted tissue distribution with high levels of expression in cerebellar Purkinje neurons and cochlear hair cells, and in mammary glands during lactation^15^,^16^. In the rat and mouse mammary gland from lactating animals, PMCA2 is the predominant isoform^6^,^17^.

The role of PMCA2 in lactation is evidenced by the phenotype of PMCA2 null mice, which in addition to harboring defects in hearing and balance^18^, produce milk with significantly lower levels of calcium ions^4^. Additionally, weaning-associated *PMCA2* suppression is a critical regulator of mammary epithelial apoptosis during involution^19^. Despite the important role of PMCA2 in the rodent mammary gland, there have been no studies of PMCA2 in the context of changes associated with lactation in humans, and there have been only limited studies of PMCA2 in the context of human breast cancer. *PMCA2* mRNA levels are elevated in some breast cancer cell lines^20^, and a tissue microarray (TMA) study suggested that high expression of PMCA2 protein predicts poor survival in patients under 50 years of age, and is associated with HER2-positive disease^19^. In terms of functional evidence, exogenous expression of PMCA2 in T47D breast cancer cells reduces their sensitivity to cell death mediated by the calcium ionophore ionomycin^19^, and disruption of PMCA2’s interaction with calcineurin can trigger apoptosis in a variety of breast cancer cell lines^21^. However, the biological role of PMCA2 in breast carcinogenesis is generally not well understood, and the breast cancer subtypes where it might be most important and its potential utility as a therapeutic target are also still unclear.

In this study, we assessed the expression of PMCA2 protein in normal human breast tissue with histologic evidence of lactational change, and the association between plasmalemmal PMCA2 protein and mRNA levels were assessed against histopathologic indicators and molecular subtype markers in breast cancer. We also evaluated the consequences of *PMCA2* silencing on the proliferation of MDA-MB-231 breast cancer cells and their sensitivity to doxorubicin, an anthracycline chemotherapy frequently used to treat breast cancer.

## Results

### PMCA2 expression in human breast tissue exhibiting lactational remodeling and malignant transformation

**Figure 1:**
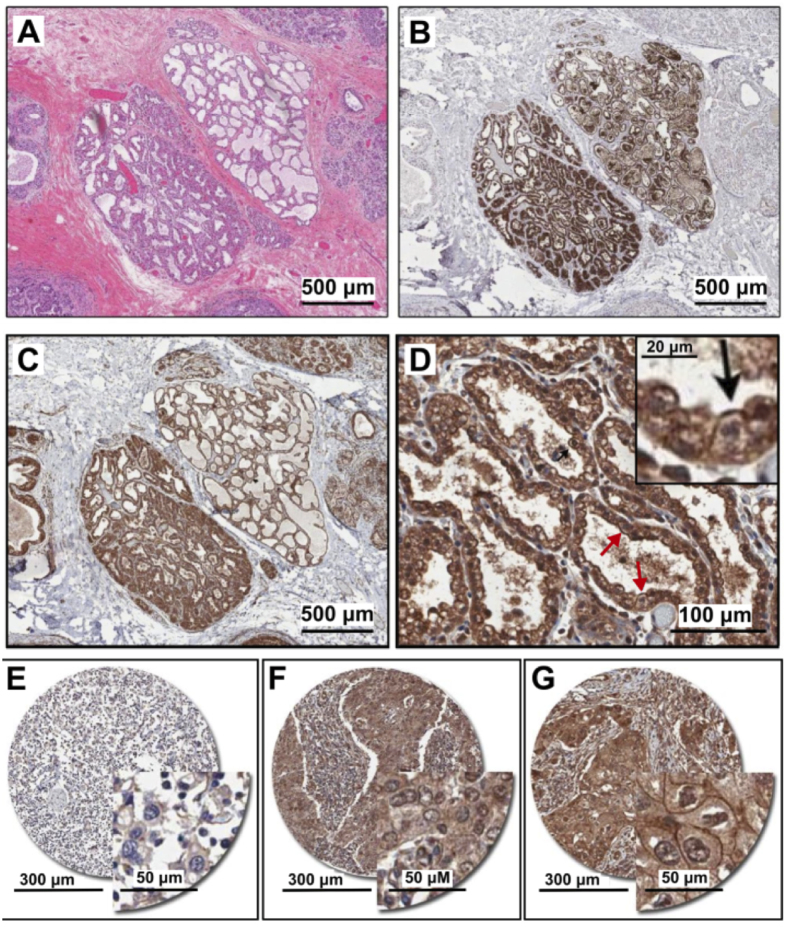
PMCA2 expression in human breast tissue with lactational change and in breast cancer tissues. (**A**–**D**) Human breast sections with lactational change; (**A**) hematoxylin and eosin staining; (**B**) β-casein staining showing accumulation of milk protein in the lumen; (**C**,**D**) PMCA2 staining showing PMCA2 accumulation on apical membranes of epithelial cells with lactational change. (**E**–**G**) Examples of PMCA2 staining in breast cancer tissues; (**E**) PMCA2-negative cancer, showing light diffuse brown background staining, (**F**) More intense PMCA2 staining in the cytoplasm with no plasma membrane localization, these were also defined as PMCA2-negative cancer in our analysis, (**G**) Clear PMCA2 staining on the plasma membrane, these were defined as PMCA2-positive cancers (PMCA2-PM+) given the localization of the PMCA2 plasma membrane Ca^2+^ pump on the plasma membrane of these breast cancer cells. Original magnification 3× (**A–C**), 20× (**D**), 5× main images and 20× for insert (**E**–**G**).

Elevated PMCA2 is a feature of mammary glands from lactating mice^2^,^22^, however, PMCA2 expression has not been assessed in human breast tissue undergoing lactational change. Therefore, we used unique tissue specimens from a breast cancer patient in the third trimester of pregnancy to investigate PMCA2 expression in histologically normal glandular tissue in the context of lactational remodeling (morphological changes and positive β-casein staining are shown in Fig. 1A,B). Positive PMCA2 staining was observed on the plasma membranes of the epithelial cells, but not the surrounding stromal cells (Fig. 1C,D). The magnified image (Fig. 1D, arrows and insert) shows elevated PMCA2 expression on the luminal membrane compared to the basal membrane, consistent with a role for PMCA2 in the direct transport of Ca^2+^ into milk.

The possible pathological role of PMCA2 was assessed in human breast cancer samples. TMAs comprising 96 breast tumors in duplicate were assessed for PMCA2 expression by IHC. The tumors were mostly histological grade 3 invasive ductal carcinomas (Supplementary Table 1). Figure 1E–G show examples of the three types of staining observed: no staining, cytoplasmic staining or membranous staining. Since PMCA2 is a plasma membrane Ca^2+^-transporter^23^, tumors with cytoplasmic but not membrane staining were classified as negative in our analysis. In total, 9/96 (~9%) of invasive tumors analyzed were PMCA2 plasma membrane positive (PMCA-PM+). We investigated associations between PMCA2 plasmalemmal expression and key prognostic indicators (histological grade, estrogen receptor (ER), progesterone receptor (PR) and HER2 status). A significant correlation between PMCA2-PM+ staining and the common breast cancer pathological markers, tumor grade, ER, PR or HER2 status was not observed (Table 1; Fisher’s exact tests, *P* > 0.05). However, we found a relationship with HER2-positivity (Table 1), with 8/9 of the PMCA2-PM+ cases also HER2+ according to clinical diagnostic criteria (>6 copies of the *ERBB2* gene by SISH). PMCA2 membrane expression is not particularly frequent in breast cancer and so in a cohort of 96 tumors this association did not reach statistical significance (Fisher’s exact *P* = 0.077), nevertheless given that the same trend was observed by others in a separate cohort^19^ this relationship could illuminate aspects of the biology underlying PMCA2 function and/or behavior of HER2+ breast tumors.

### PMCA2 mRNA is significantly enriched in the Basal breast cancer molecular subtype where is it associated with survival

**Figure 2:**
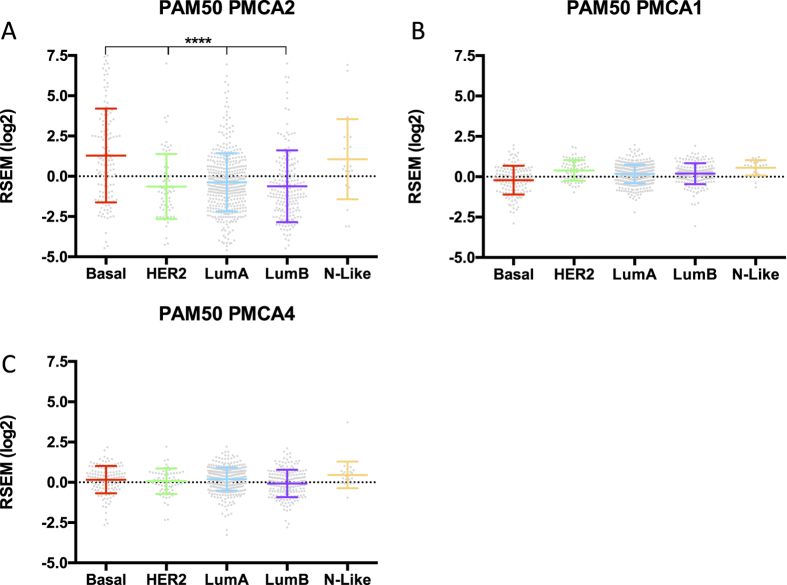
PMCA2 is expressed most highly in Basal-like breast cancer. Log2 row-mean centred RSEM data sourced from the TCGA consortium. (**A**–**C**), expression for PMCA2, PMCA1 and PMCA4, respectively. Significance was found through a one-way ANOVA with a Tukey’s multiple comparisons test, ****P < 0.0001 between the Basal-like tumors and HER2, LumA and LumB.

**Figure 3:**
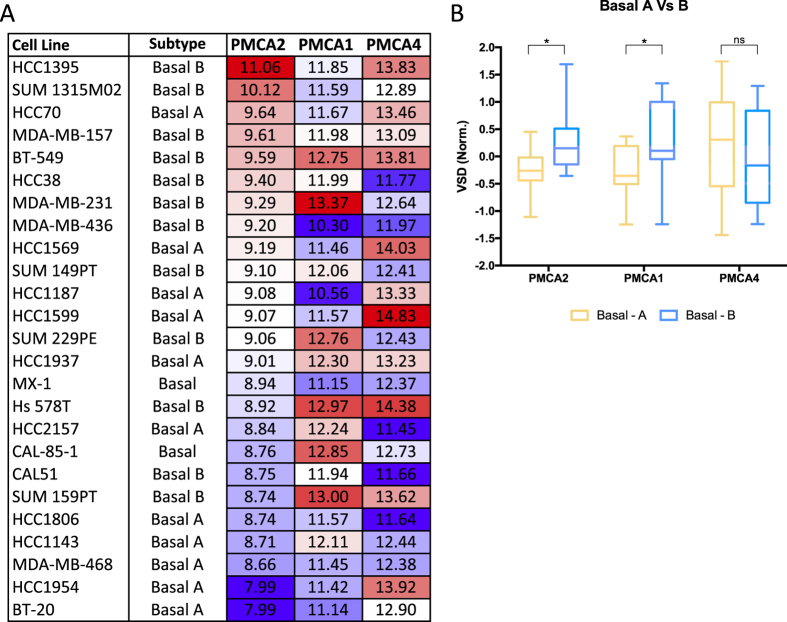
PMCA2 is dynamically expressed in Basal breast cancer cell lines. Expression values are derived from Variance Stabilized Data (VSD, a measure of expression coined by Klijn *et al.*^53^. (**A**) log2 VSD expression data in order of highest to lowest expressing cell line based on PMCA2/ATP2B2. Each column is colour-coded red for high and blue for low expression. (**B**) Average expression of Basal A vs Basal B cell lines. Significance was found using a two-tailed unpaired T test, *P < 0.05.

**Figure 4:**
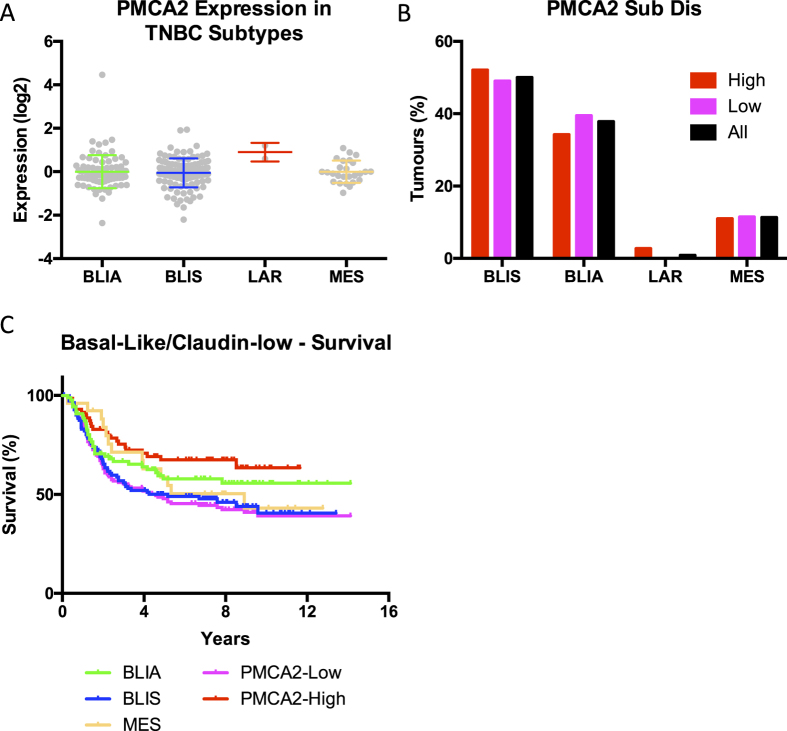
PMCA2 expression stratifies Basal breast cancers independent of TNBC subtypes. (**A**) Basal-like and Claudin-low breast cancers^51^ were assigned a TNBC subtype based on expression gene expression patterns from^52^; Basal-like Immune Activated (BLIA), Basal-like Immune Suppressed (BLIS), Luminal AR (LAR) and Mesenchymal (MES). (**B**) Distribution of TNBC-Subtypes in low and high expressing PMCA2 tumors and across all Basal-like and Claudin-low tumors. No significance was found between PMCA2 groupings via a χ2 test, P-value = 0.825. (**C**) Relapse-free-survival (RFS) for each of the TNBC-Subtypes compared to PMCA2 expression groupings. No significance was found between BLIA and BLIS tumors (Log-rank P-value 0.1488), however PMCA2 groupings were significant with a hazard ratio of 0.50 (95% CI 0.36 – 0.80) and P-value = 0.0024 comparing high expression to low.

*PMCA2*, *PMCA1 and PMCA4* mRNA levels were compared in breast cancer molecular subtypes^24^ from RSEM data from the TCGA consortium (Fig. 2A–C). Consistent with the IHC data presented above, there were individual breast cancers with relative high levels of PMCA2 (ATP2B2) in all of the molecular subtypes (Fig. 2A), this was not seen for PMCA1 (ATP2B1) or PMCA4 (ATP2B4) (Fig. 2B,C). PMCA2 levels were, however, significantly enriched in the Basal molecular phenotype compared to HER2, Luminal A and Luminal B. Assessment of PMCA2 levels in Basal breast cancer cell lines identified PMCA2 as the minor isoform at the mRNA level in all basal breast cancer cell lines, with a trend for cell lines with higher levels of PMCA2 to be identified as Basal B (Fig. 3A). Indeed, PMCA2 levels were significantly greater in Basal B breast cancer cell lines compared to Basal A, a trend which was also observed for PMCA1 but not PMCA4 (Fig. 3B). In contrast to basal breast cancer cell line differences, PMCA2 levels were not significantly different between the recently defined triple-negative breast cancer (TNBC) molecular subtypes, BLIS, BLIA, LAR and MES (Fig. 4A) and there was no significant distribution of tumor subtypes in the low and high PMCA2 expression groups (Fig. 4B). However, assessment of patient survival in TNBC, identified PMCA2 levels as positively associated with patient survival, this stratification was more pronounced than the stratification by the defined molecular subtypes in TNBC; BLIA, BLIS and MES (Fig. 4C). The positive association between PMCA2 levels and survival in Basal breast cancers was also observed in three different patient cohorts and this positive association was not consistently seen for PMCA1 or PMCA4 (Supplementary Tables 2–4). PMCA2 mRNA was significantly elevated in specific breast cancers in both HER2 and Basal molecular subtypes, although within each subtype there was clear variation (Supplementary Fig. 1), this variance within Basal breast cancers may be the cause of the association between PMCA2 and survival in this subtype. Correlation analysis demonstrated a positive and significant correlation between PMCA2 and the Basal marker EGFR across all breast cancers, however, within the Basal subtype this association was negative (Supplementary Fig. 1).

### PMCA mRNA levels in MDA-MB-231 Basal-like breast cancer cells

**Figure 5:**
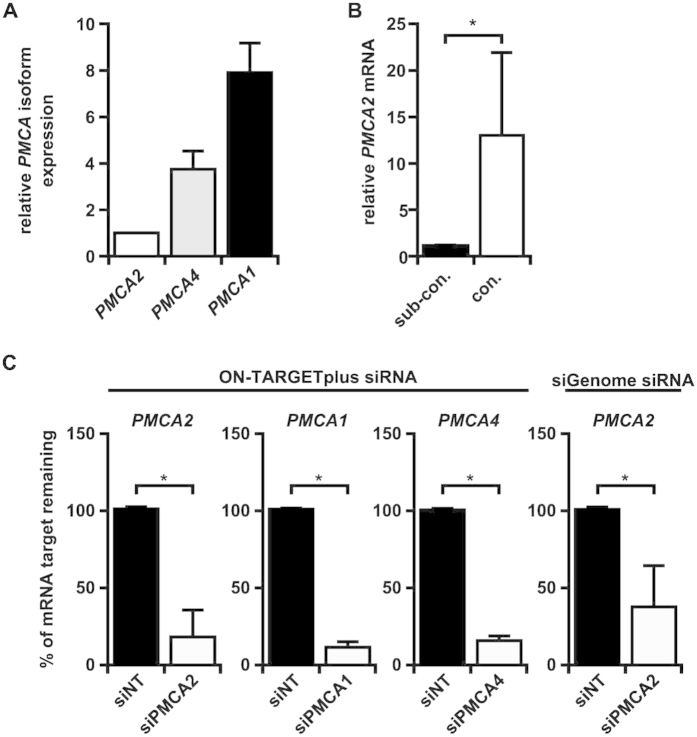
*PMCA* mRNA levels in MDA-MB-231 breast cancer cells and silencing of *PMCA* isoforms in MDA-MB-231 cells. (**A**) *PMCA* isoform mRNA levels in confluent MDA-MB-231 cells relative to *PMCA2* mRNA. (**B**) *PMCA2* mRNA levels in sub-confluent (sub-con.) and confluent (con.) MDA-MB-231 cells. (**C**) *PMCA2*, *PMCA1* and *PMCA4* siRNA mediated silencing, 120 h after transfection with non-targeting siRNA (siNT), *PMCA2* siRNA (siPMCA2), *PMCA1* siRNA (siPMCA1) or *PMCA4* siRNA (siPMCA4). The data are mean ± SD (*n* = 3) and are from three independent experiments, *P < 0.05, unpaired t*-*test.

Given the focus of past studies of PMCA2 on luminal-like breast cancer cells, PMCA2 was assessed in MDA-MB-231 cells that are a representative Basal B breast cancer cell line. *PMCA2* mRNA was detected in MDA-MB-231 (Basal-B/Claudin-low molecular subtype^25^) by real-time RT-PCR. Its expression was >3-fold lower than *PMCA1* and *PMCA4* isoforms (Fig. 5A), which contrasts with rodent models of lactation where PMCA2 is the predominant isoform^6^,^17^ and was consistent with our RNAseq cell line data of MDA-MB-231 and other Basal breast cancer cell lines. *PMCA2* mRNA expression was significantly higher in confluent compared to sub-confluent cells (Fig. 5B). PMCA2 protein analysis in this cell line, suggested low levels of PMCA2 protein or a lack of full length PMCA2 protein in MDA-MB-231 cells, despite confidence in the antibody and techniques used (e.g. PMCA2 was readily detectable in human tissue; Fig. 1D,G). This may relate to the transient expression of full length PMCA2 protein during specific cell cycle stages and/or the potential for a PMCA2 fragment to be expressed which has been reported to have biological activity.

### Silencing PMCA2 inhibits proliferation of breast cancer cells

**Figure 6:**
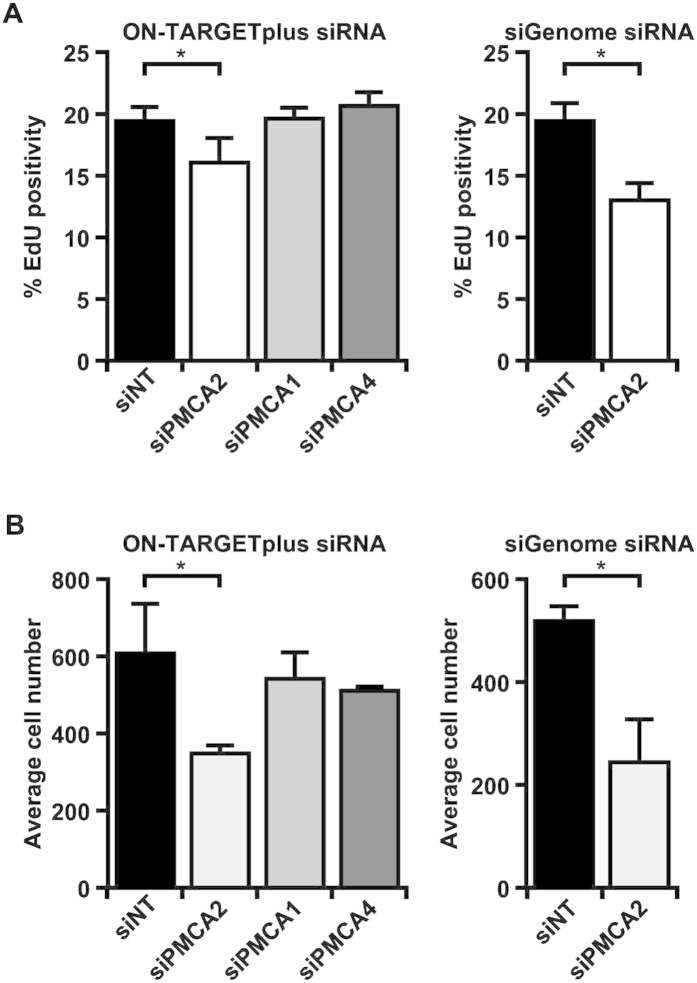
Silencing *PMCA2* in MDA-MB-231 breast cancer cells inhibits the percentage of cells in S-phase and reduces cell number. (**A**,**B**) Silencing *PMCA2* for 120 h using ON-TARGETplus siRNA and siGenome siRNA inhibits (**A**) the percentage of EdU-positive cells and (**B**) cell number. Data are mean ± SD (*n* = 3) from 3 independent experiments, *P < 0.05, one-way ANOVA, Tukey’s post hoc test (left panel) or unpaired t-test (right panel).

Exogenous expression of PMCA2 in luminal T47D breast cancer cells protects against ionomycin-mediated death^19^. Here, we assessed the effect of silencing endogenous PMCA2 on the proliferation of basal-like MDA-MB-231 cells, which express elevated *PMCA2* mRNA compared to non-malignant breast cell lines^26^. PMCA isoform expression was silenced using siRNA, validating knockdown by real-time RT-PCR (Fig. 5C). PMCA2 silencing was not associated with a significant compensatory change in PMCA1 or PMCA4 (Supplementary Fig. 2). Assessment of proliferating cells using EdU staining showed that *PMCA2* silencing reduced the percentages of cells in S-phase by 17% and 33% (*P* < 0.05; Fig. 6A), with corresponding decreases in total cell number of 43% and 53%, using two separate siRNAs (Fig. 6B). In contrast, silencing *PMCA1* or *PMCA4* had no significant effect on cell cycle progression rate or cell number (Fig. 6).

### Effects of PMCA2 silencing combined with cytotoxic chemotherapy on Ca^2+^ signaling and proliferation

**Figure 7:**
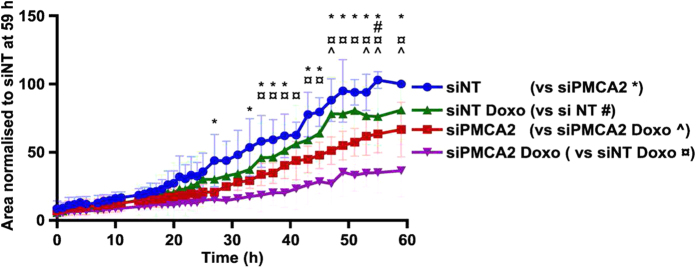
*PMCA2* silencing enhances the inhibitory effects of doxorubicin on MDA-MB-231 breast cancer cell proliferation. Cells were transfected with non-targeting siRNA (siNT) or *PMCA2* siRNA (siPMCA2) for 48 h, and then pulse treated with doxorubicin (Doxo, 20 nM) for 24 h. Cell area was assessed for 59 h. Data are mean ± SD (*n* = 4) from 4 independent experiments, statistical significance was assessed using two-way ANOVA with Tukey’s post hoc test for each time point, *P < 0.05, for siNT vs siPMCA2; ^#^*P* < 0.05, siNT vs siNT Doxo; ^¤^*P* < 0.05, siNT Doxo vs siPMCA2 Doxo; ^^^*P* < 0.05, siPMCA2 vs siPMCA2 Doxo.

The use of rational combination therapies reduce the likelihood that tumors will develop therapeutic resistance^27^, and that the patient will experience toxic side effects^28^. Others have shown PMCA2 deficiency leads to increased sensitivity to Ca^2+^-induced apoptosis^19^, and we hypothesized that *PMCA2* suppression may enhance the effects of cytotoxic chemotherapy on breast cancer cells. We tested this by assessing doxorubicin efficacy in the si*PMCA2* MDA-MB-231 model. Consistent with the anti-proliferative effects identified in high-content analysis (Fig. 6), *PMCA2* silencing attenuated MDA-MB-231 cell proliferation (Fig. 7; *P < 0.05). A pulse treatment with a very low dose of doxorubicin (20 nM) had negligible impact on the proliferation of MDA-MB-231 cells transfected with control non-targeting siRNA (NT siRNA; Fig. 7, ^#^*P* < 0.05). However, doxorubicin promoted the anti-proliferative effects of *PMCA2* silencing (Fig. 7, ^^^*P* < 0.05) and inhibited cell proliferation more effectively than *PMCA2* silencing or doxorubicin treatment alone (Fig. 7). No morphological signs of promotion of cell death with *PMCA2* silencing were observed (Supplementary movies).

**Figure 8:**
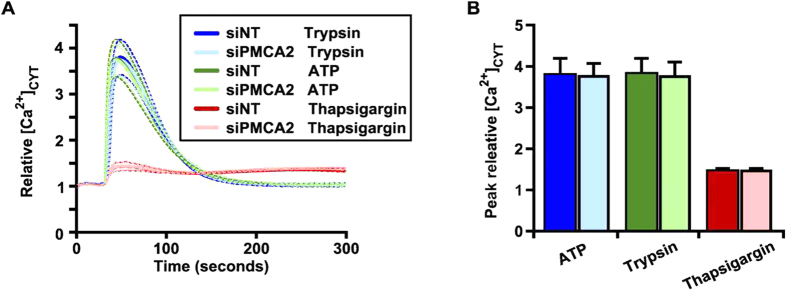
Effect of doxorubicin and *PMCA2* silencing on Ca^2+^ signaling in MDA-MB-231 cells. Cells were transfected with non-targeting siRNA (siNT) or *PMCA2* siRNA (siPMCA2) for 48 h, and pulse treated with doxorubicin (20 nM) for 24 h and then incubated for an additional 24 h. (**A**) Relative [Ca^2+^]_CYT_ response to trypsin (100 nM), ATP (1 mM) or thapsigargin (2 μM) in MDA-MB-231 cells treated with siNT or siPMCA2. Relative [Ca^2+^]_CYT_ (solid line) ± SD (dotted line). (**B**) Mean peak relative [Ca^2+^]_CYT_ (*n* = 3) ± SD from 3 independent experiments.

Given the potential for PMCA2 to contribute to changes in or doxorubicin sensitivity via global or local effects on calcium signaling or even other pathways^21^ we assessed global Ca^2+^ signaling responses of *PMCA2*-silenced and non-silenced doxorubicin-treated MDA-MB-231 cells to three agents known to produce transient increases in intracellular free Ca^2+^ ([Ca^2+^]_CYT_): ATP, trypsin and thapsigargin (an inhibitor of the endoplasmic reticulum Ca^2+^ ATPase). We found that regardless of the stimulus used, increases in [Ca^2+^]_CYT_ in doxorubicin-treated cells were not altered by *PMCA2* silencing, in terms of both the nature of the recovery of [Ca^2+^]_CYT_ (Fig. 8A) or the maximum [Ca^2+^]_CYT_ level achieved (Fig. 8B). These data suggest that the effects of PMCA2 silencing on cell proliferation is independent of effects on global levels of [Ca^2+^]_CYT._

## Discussion

The altered expression of specific Ca^2+^ channels is a characterizing feature of many cancers^7^,^8^,^9^. These include enhanced expression of specific isoforms of transient receptor potential (TRP)^29^,^30^,^31^ and Orai Ca^2+^ permeable ion channels^32^,^33^, as well as voltage^34^,^35^,^36^ and ligand gated Ca^2+^ channels^37^,^38^,^39^. Although not as widely characterized, altered expression of particular isoforms of p-type Ca^2+^-ATPase family members is associated with specific cancer subtypes, for example, elevated levels of SPCA1 in basal-like breast cancers^40^ and of SERCA2 in colorectal cancers^41^,^42^. The identification of *PMCA2* mRNA in breast cancer cell lines^20^,^26^, PMCA2 protein in clinical breast cancer specimens^19^, and a role for PMCA2 in the transport of Ca^2+^ into milk during lactation^4^, highlight the relevance of this p-type ATPase in the context of human breast cancer and the physiology of the human breast.

Our finding that PMCA2 is expressed at the apical membrane of luminal epithelia in the pre-lactational human breast is consistent with data from rodent models demonstrating that PMCA2 is a key pump responsible for the efflux of Ca^2+^ from the maternal compartment into milk. In the breast cancer cohort assessed in this study, 9/96 tumors expressed PMCA2 in the tumor cell plasma membrane. Consistent with VanHouten *et al.*^19^, we found a positive association between PMCA2 expression and HER2 status, with eight out of the nine PMCA2 membrane-positive cases classified HER2+. This relationship did not reach statistical significance, owing largely to the size of the PMCA2+ subgroup in our study (*n* = 9 cases). A key difference between our study and VanHouten’s was that we assessed the subcellular localization of PMCA2. We analyzed membrane-associated PMCA2 as a categorical variable, whereas the previous study used digital scoring to quantify overall tumor cell positivity as a continuous variable. This is an important distinction given that the membrane residence time of ion pumps is dynamic and often tightly regulated, and subcellular compartment-specific expression of PMCA2 alternative splice isoforms^43^ has not been thoroughly investigated.

Our investigation of PMCA2 levels in molecular breast cancer subtypes supported our IHC data of high levels of PMCA2 across different breast cancer subtypes. Specific breast cancers of the Basal, Luminal A, Luminal B and HER2 molecular subtypes had high levels of PMCA2, this was not as obvious for PMCA1 and PMCA4. However, this large cohort identified that PMCA2 mRNA levels were significantly higher in basal breast cancers overall compared to Luminal A, Luminal B and HER2 subtypes. The absence of any differences in PMCA1 and PMCA4 in the different molecular subtypes reinforces the potential unique roles of the PMCA2 isoform in the breast in both lactation and in breast cancer. Although PMCA2 levels were higher in Basal B vs Basal A breast cancer cell lines, PMCA2 levels were not different amongst the recently identified TNBC molecular subtypes BLIS, BLIA, LAR and MES. However, PMCA2 levels were highly correlated with patient survival in triple negative breast cancers and basal breast cancers, which was seen across multiple cohorts. In these cases, high levels of PMCA2 were associated with better patient survival. This is in contrast to a previous report of Oncomine cDNA microarray data in only patients under the age of 50, which found a negative association between high PMCA2 levels and survival^19^. Our identified relationship between high levels of PMCA2 mRNA and patient survival in basal breast cancers, may represent an ability for PMCA2 to identify less aggressive basal breast cancers and signify that PMCA2 overexpression is a not a driver in breast cancer. The potential dichotomy in PMCA2 levels between subtypes and its correlation with survival in the basal subtype is exemplified by the very different association between EGFR and PMCA2 in all breast cancers (positive correlation) versus the basal subtype (negative correlation). Hence, within basal breast cancers, PMCA2 may associate with characteristics of better prognosis which may make it a biomarker for good survival. This association does not exclude the potential of PMCA2 as a drug target in some breast cancer cells, either via the previously proposed mechanism of promotion of apoptosis through PMCA2 inhibition^19^ and/or the anti-proliferative effects of PMCA2 inhibition identified in MDA-MB-231 cells in this study. These complex relationships and associations could be further explored by detailed comparisons of the consequences of PMCA2 silencing in different cell-lines as well as different patient derived xenograft models.

Previous studies of PMCA2 in human breast cancer cells have focused on the role of this Ca^2+^ pump in protection against cell death mediated by agents that produce sustained increases in [Ca^2+^]_CYT_^19^ or its role in apoptosis regulation through interactions with calcineurin^21^. However, calcium signaling also plays a vital role in cell cycle regulation in cancer cells^7^ and global inhibition of PMCA expression reduces the proliferation of ER+, luminal-like MCF-7 breast cancer cells^44^. In these studies, PMCA2 was the only PMCA isoform associated with proliferation of basal-like MDA-MB-231 cells, despite the lower expression of this pump relative to other isoforms. The lack of any effect of *PMCA1* silencing on cellular proliferation may seem surprising given that this isoform is the predominant regulator of global [Ca^2+^]_CYT_ in MDA-MB-231 cells^45^. However, Curry *et al.* demonstrated that despite only modest effects on global [Ca^2+^]_CYT_ with *PMCA4* silencing, *PMCA4* but not *PMCA1* silencing augmented apoptosis mediated by the Bcl-2 inhibitor, ABT-263, likely through selective effects on Ca^2+^ dependent NFkB activity^45^. The ability of *PMCA2* to regulate MDA-MB-231 proliferation may also be due to localized specific regulation of Ca^2+^ dependent transcription factors involved in proliferation. However, other mechanisms are also possible. For example, the pronounced increase of *PMCA2* mRNA in MDA-MB-231 cells with increasing confluence *in vitro* may reflect dynamic expression of PMCA2 at critical stages of the cell cycle, such that silencing of *PMCA2* has pronounced effects only at specific stages of cell division. Moreover, the ability of specific regions of PMCA2 to interact with calcineurin^21^ suggests that the entire intact PMCA2 protein may not be required to have a functional consequence in at least some breast cancer cells.

Our data raise the possibility that PMCA2 depletion or inhibition could be a chemo-sensitizing strategy in some breast cancer cells^21^. On the background of PMCA2 deficiency, we found that a low dose of doxorubicin was sufficient for pronounced inhibition of MDA-MB-231 proliferation *in vitro*, suggesting tumor-targeted PMCA2 depletion or inhibition may allow the use of doxorubicin doses associated with a better side effect profile in these cells.

## Conclusions

These data provide further evidence for an important role of PMCA2 in calcium transport during human lactation, and the expression of PMCA2 in a significant percentage of breast cancers. PMCA2 function does not appear to be restricted to the regulation of cell death pathways in breast cancer cell lines, and may regulate other hallmarks of cancer including sustained cellular proliferation^46^. Targeting PMCA2 to reduce breast tumor cell proliferation and increase sensitivity to cytotoxic chemotherapy is a strategy worth further investigation.

## Methods

### Human clinical samples

Formalin-fixed, paraffin-embedded (FFPE) samples of histologically normal human breast tissue exhibiting pregnancy-induced lactational change were obtained from Pathology Queensland. This patient presented with breast cancer in the third trimester of pregnancy, and underwent a wide local excision procedure. Histopathologic diagnostic assessment revealed a grade 3 invasive ductal carcinoma of no special type (negative for estrogen, progesterone and human epidermal growth factor receptors (‘triple-negative’)), associated with high-grade ductal carcinoma *in situ* and lymph node metastases. Immunohistochemistry (IHC) was performed on glandular tissue in the specimen that exhibited hyperplasia, but no evidence of *in situ* or invasive disease. Physiological hyperplasia is an expected feature in the pre-lactation breast.

For breast cancer studies, tissue microarray (TMA) sections containing duplicate tissue cores (0.6 mm) from 96 tumors (supplementary data, Table 1) were constructed using archival FFPE blocks from Pathology Queensland^47^. The retrospective analysis of archival human clinical samples in this study was approved the human research ethics committees at the Royal Brisbane and Women’s Hospital and The University of Queensland (UQ 2005000785; RBHW 2005/022). All studies were conducted in accordance with institutional approved guidelines.

### Immunohistochemistry

Tissue sections (4 μm) were deparaffinized, rehydrated, washed and heated in 0.01 M citrate buffer (pH 6) at 125 °C for 5 min and at 90 °C for 10 min in a decloaking chamber (Biocare Medical). Sections were stained using the rabbit anti-PMCA2 ATPase polyclonal antibody (1:300; PA1-915 Thermo-Fisher Scientific) or β-casein monoclonal antibody (1:100; sc-53189, Santa Cruz), and the MACH-1 Universal HRP-Polymer Detection Kit (Biocare Medical) according to the manufacturer’s instructions. Nuclei were counterstained with hematoxylin using a Varistain Gemini ES Automated Slide Stainer (Thermo Fisher Scientific). The negative and positive controls were no primary antibody and cerebellar tissue, respectively. Stained tissue sections were scanned at 20× magnification using a ScanScope XT Digital Slide Scanner (Aperio), and evaluated by a blinded pathologist (LdS) using the following criteria: (1) positive: intense plasma membrane staining with or without cytoplasmic staining; (2) negative: cytoplasmic or no staining (since PMCA2 is a plasma membrane Ca^2+^-transporter).

### Breast Tumor Expression Analysis

Analysis of RNA-Seq for PMCA2 (ATP2B2), PMCA1 (ATP2B1) and PMCA4 (ATP2B4) and breast cancer molecular markers (KRT5, ERBB2, MK167, FOXM1, AURKA, EGFR, PGR, ESR1, FOXA1 and TFF) utilized the publically available TCGA (The Cancer Genome Atlas) dataset accessible through cbioportal.org^48^. This dataset consisted of a total 1100 tumors, of these 845 were classified into the PAM50 intrinsic molecular subtypes (Basal-like (140), HER2-enriched (67), Luminal A (420), Luminal B (194) and Normal-like (24) (as postulated by Perou and Sorlie 24,49 by TCGA). The RNA-Seq data from the TCGA were processed by the RSEM software^50^ and was then log2 transformed and mean-centered by gene (row). Tumor samples were then hierarchically clustered based on their gene expression profiles using Multiple Experiment Viewer (MeV, PMID: 9843981, http://www.tm4.org/mev.html) via a Manhattan average-linkage based algorithm. Gene Pearson’s correlations and their corresponding P-values were determined in Microsoft Excel (Version 15.19.1) via the ‘correl’ function. Breast tumors from the University of North Carolina (UNC) cohort^51^ were assigned into one of the triple-negative breast cancer (TNBC) subtypes based on a published approach^52^. The UNC cohort contains a total of 230 Basal-like and Claudin-low tumors with 115 Basal-like Immune Suppressed (BLIS), 78 Basal-like Immune Activated (BLIA), 2 Luminal androgen receptor (LAR) and 26 Mesenchymal (MES).

### Cell Line RNA-Seq

Cell line RNA-Seq was sourced from Klijn *et al.*^53^. These data have been mapped and normalized using their novel methodology of Variance Stabilized Data (VSD). Gene expression (Log2 normalized VSD) values were extracted from this dataset for all available Basal-like breast cancer cell lines for PMCA2, PMCA1 and PMCA4 and displayed as shown without any further normalization.

### Assignments of PMCA expression groups

Both the UNC^51^ and Veridex (VDX)^54^ cohorts utilize microarray expression. Affymetrix probe sets for VDX genes were combined and averaged and used in downstream analysis (PMCA2/ATP2B2 204685_s_at, 211586_s_at and 216120_s_at, PMCA1/ATP2B1 209281_s_at, 212930_at and 215716_s_at and PMCA4/ATP2B4 205410_s_at, 212135_s_at and 212136_at). For high and low expression groups in the UNC and VDX cohorts of Basal-like and/or Claudin-low tumors, receiver-operator characteristic (ROC) curves were produced for PMCA2, PMCA1 and PMCA4 expression against survival outcome (relapse-free survival (RFS) and distant-metastasis-free survival (DMFS)). These curves were produced using the software MedCalc (www.medcalc.org), with optimal values used to call expression cutoff points. Percentile cutoffs are reported in supplementary Tables.

### Survival Analysis

Survival analysis was performed in both UNC and VDX cohorts and with the online tool Kaplan-Meier Plotter^55^. For the UNC and VDX cohorts, RFS and DMFS, respectively, were stratified on the basis of PMCA2, PMCA1 and PMCA4 expression groups as described above. Univariate Cox proportional-hazards regression was carried out using MedCalc with results reported in the supplementary data. The Kaplan-Meier curve was produced using Prism software with Log-rank hazards ratios and P-values reported with each figure. Survival analysis from the Kaplan-Meier Plotter cohort of breast tumors was done using the ‘Auto-select best cutoff,’ feature on the website, which analyses the median, tertile and quartile cutoffs for the more significant P-value.

### Cell culture

MDA-MB-231 cells (obtained from ATCC) were cultured in high-glucose Dulbecco’s modified Eagle’s medium (DMEM, Sigma-Aldrich) supplemented with 10% FBS and L-Glutamine (4 mmol/L) (Sigma-Aldrich) at 37 °C with 5% CO_2_. The cells were cultured for less than 10 passages before experimentation and were monitored for morphological changes. STR profiling is regularly performed to authenticate the cell line using the StemElite ID Profiling Kit (Promega) at QIMR Berghofer (the last relevant test for these studies was performed February 2014, Brisbane, Australia as experiments were finalized prior to this date). Cells were tested 6-monthly for mycoplasma (MycoAlert Assay, Lonza).

### Transfection with siRNA

MDA-MB-231 cells seeded in 96-well plates (5 × 10^3 ^cells/well) were transfected with Dharmacon ON-TARGET*plus* SMARTpool siRNA or siGENOME SMARTpool siRNA (GE Healthcare) at a final concentration of 100 nmol/L using 0.1 μL/well DharmaFECT 4 according to the manufacturer’s instructions. The following human ON-TARGET*plus* siRNAs were transfected: non-targeting (siNT, D-001810-10-05), PMCA1 (siPMCA1, L-006115-00-0005), PMCA2 (siPMCA2, L-006116-00-0005) and PMCA4 (siPMCA4 L-006118-00-0005). We also used the following human siGENOME siRNAs: non-targeting (siNT, D-001206-14-05) and PMCA2 (siPMCA2, M-006116-00-0005). Knockdown of *PMCA2*, *PMCA1* or *PMCA4* was confirmed by real-time RT-PCR at 120 h post-transfection.

### Real-time RT-PCR

RNA was isolated as previously described^26^ and reverse transcribed using the Omniscript RT kit (Qiagen). Real-time RT-PCR was performed using Taqman Fast Universal PCR Master Mix and gene expression assays: PMCA1 (Hs00155949_m1), PMCA2 (Hs00155975_m1), PMCA4 (Hs00608066_m1) with 18S rRNA as an input control (4319413E). Reactions were performed using StepOnePlus system (Applied Biosystems) with universal cycling conditions. Relative mRNA expression levels were determined using the comparative C_T_ method^56^.

### EdU incorporation assays: cell proliferation and cell cycle

Total MDA-MB-231 cell numbers and the proportion in S-phase of the cell cycle were assessed as previously described^57^. Briefly, 120 h after siRNA transfection, cells were treated with EdU (10 mmol/L), fixed with 3.7% formaldehyde, and permeabilized with 0.5% Triton X-100. The Click-iT reaction cocktail (Alexa Fluor 555; Life Technologies) was incubated with the cells, followed by DAPI (4′6-diamidino-2-phenylindole; 400 nmol/L). The cells were imaged with the ImageXpress® Micro (Molecular Devices) automated epiflourescent microscope (10× objective). The DAPI and EdU stained cells were detected as described previously^57^, and analysis was performed using the multiwavelength cell scoring application module (MetaXpress).

### Treatment with a cytotoxic to assess cell proliferation and intracellular-free Ca^2+^ [Ca^2+^]_CYT_

MDA-MB-231 cells were transfected with siRNAs for 48 h as described above. Cells were pulse treated with Doxorubicin (Doxo, 20 nM) for 24 h, cells were washed twice with Phosphate Buffered Saline (PBS), and the media was replaced with standard growth media. To assess cell proliferation, the total area of the cells was assessed for a period of 59 h using a kinetic imaging system, IncuCyte ZOOM (Essen Bioscience). Intracellular-free Ca^2+^ [Ca^2+^]_CYT_ was assessed 48 h after doxorubicin treatment using the BD PBX Calcium Assay Kit (BD Biosciences^58^) as described previously^57^ with minor modifications. Briefly, cells were loaded with the Calcium Indicator, 5% PBX Signal Enhancer and probenecid (500 μmol/L) in physical salt solution (PSS; with 1.8 mmol/L CaCl_2_) for 1 h at 37 °C. The loading solution was replaced with PSS containing nominal Ca^2+^, 5% PBX Signal Enhancer and probenecid (500 μmol/L). Fluorescence was assessed with an excitation intensity of 470–495 nm and a 515–575 nm emission filter using a Fluorescence Imaging Plate Reader (FLIPR)^TETRA^ (Molecular Devices). Fluorescence was normalized to the baseline fluorescence and expressed as ‘relative [Ca^2+^]_CYT_’.

### Statistical Analysis

Statistical associations between PMCA2 and breast cancer prognostic indicators were evaluated using the Fisher’s exact test. Statistical significance for the remaining data was assessed as described in individual figure legends. All statistical analyses were performed using GraphPad Prism (version 6.04 for Windows and version 6.0f for Mac OS X, GraphPad Software, Inc.).

## Additional Information

**How to cite this article**: Peters, A. A. *et al.* The calcium pump plasma membrane Ca^2+^-ATPase 2 (PMCA2) regulates breast cancer cell proliferation and sensitivity to doxorubicin. *Sci. Rep.* **6**, 25505; doi: 10.1038/srep25505 (2016).

## Supplementary Material

Supplementary Information

Supplementary Video 1

Supplementary Video 2

## Figures and Tables

**Table 1:** Associations between PMCA2 expression and prognostic indicators in breast cancer.

Histopathologic indicators	PMCA2-PM positive *n* (%)	PMCA2 negative *n* (%)	Fisher’s exact *p* value
Histological grade 1 and 2 3	2 (12) 7 (9)	15 (88) 68 (91)	0.67
ER Positive Negative	1 (5) 8 (11)	20 (95) 66 (89)	0.678
PR Positive Negative	0 (0) 9 (11)	13 (100) 73 (89)	0.353
HER2 Positive Negative	8 (14) 1 (3)	48 (86) 38 (97)	0.077
All tumors	9 (9)	87 (91)	

(ER, estrogen receptor; PR, progesterone receptor; HER2, human epidermal growth factor receptor 2).
