# Supplementary material for: The calcium pump plasma membrane Ca2+-ATPase 2 (PMCA2) regulates breast cancer cell proliferation and sensitivity to doxorubicin
Source: Sci Rep. 2016 May 5;6:25505. doi: 10.1038/srep25505 (PMC4857793; doi:10.1038/srep25505)
Supplement: Supplementary Information [file srep25505-s1.pdf]

The calcium pump plasma membrane Ca^{2+}-ATPase 2 (PMCA2) regulates breast cancer cell proliferation and sensitivity to doxorubicin

Amelia A. Peters[1], Michael J.G. Milevskiy[2], Wei C. Lee[1], Merril C. Curry[1], Chanel E. Smart[3,4], Jodi M. Saunus[3,5], Lynne Reid[3,5], Leonard da Silva[3,5], Daneth L Marcial[1], Eloise Dray[6], Melissa A Brown[2], Sunil R. Lakhani[3,4,7], Sarah J. Roberts-Thomson[1], and Gregory R. Monteith[1,8]*

[1] School of Pharmacy, The University of Queensland, Brisbane, Queensland, 4072 Australia
[2] School of Chemistry and Molecular Biosciences, The University of Queensland, Brisbane, Queensland, 4072, Australia
[3] UQ Centre for Clinical Research, The University of Queensland, Herston, Queensland, 4006, Australia
[4] School of Medicine, University of Queensland, Brisbane, Queensland, 4072, Australia
[5]QIMR Berghofer Medical Research Institute, Cancer Genetics, Herston, Queensland, 4006, Australia
[6] School of Biomedical Sciences, Queensland University of Technology, Brisbane, Queensland, 4102, Australia
[7] Pathology Queensland, The Royal Brisbane & Women's Hospital, Brisbane, Queensland, 4006, Australia
[8] Mater Research Institute, The University of Queensland, Brisbane, Queensland, 4072, Australia

Supplementary Table 1: characteristics of the breast tumor cohort (n = 96)

**Characteristics**	**Number of patients n (%)**
**Histology**	
**Invasive Ductal Carcinoma**	89 (93)
**Mixed Ductolobular Carcinoma**	3 (3)
**Invasive Lobular Carcinoma**	2 (2)
**Others/Not specified**	2 (2)
**Tumour grade**	
**1**	2 (2)
**2**	15 (16)
**3**	75 (78)
**Not specified**	4 (4)
**ER**	
**Positive**	21 (22)
**Negative**	74 (77)
**Not available**	1 (1)
**PR**	
**Positive**	13 (14)
**Negative**	82 (85)
**Not available**	1 (1)
**HER2**	
**Positive**	56 (58)
**Negative**	39 (41)
**Not available**	1 (1)

Supplementary table 1: Characteristics of the breast tumor cohort (n=96), used to assess PMCA2 expression in table 1**.**

Supplementary Table 2: PMCA2 expression consistently stratifies TNBC, Basal-Like and Claudin-Low breast cancer.

Covariate	Cohort	Cox Proportial-Hazards Regression			ROC Optimal Cutoff	Tumor #	
		P-Value	HR	95% CI		Low	High
PMCA1 (high vs low)	**Triple Negative Breast Cancer**	0.1046	1.522	(0.919 - 2.520)	≥55.9% = High	61	49
PMCA2 (high vs low)		**0.0029**	0.379	(0.201 - 0.715)	≥67.7% = High	74	36
PMCA4 (high vs low)		**0.0166**	1.928	(1.130 - 3.292)	≥75.9% = High	83	27
PMCA1 (high vs low)	**Basal-Like and Claudin-Low**	0.149	1.325	(0.906 - 1.936)	≥65.8% = High	152	78
PMCA2 (high vs low)		**0.0041**	0.515	(0.328 - 0.808)	≥68.5% = High	157	73
PMCA4 (high vs low)		0.0772	1.524	(0.957 - 2.427)	≥84.6% = High	194	36
PMCA1 (high vs low)	**Basal-Like**	**0.005**	0.493	(0.301 - 0.806)	≥26.1% = High	36	104
PMCA2 (high vs low)		**0.031**	0.534	(0.303 - 0.942)	≥69.6% = High	97	43
PMCA4 (high vs low)		0.1002	1.594	(0.917 - 2.773)	≥29.6% = High	41	99
PMCA1 (high vs low)	**Claudin-Low**	**0.0221**	2.146	(1.120 - 4.114)	≥56.1% = High	50	40
PMCA2 (high vs low)		**0.0289**	0.418	(0.192 - 0.910)	≥67.2% = High	60	30
PMCA4 (high vs low)		0.0564	2.228	(0.983 - 5.053)	≥90.6% = High	81	9

Supplementary table 2: **PMCA2 expression consistently stratifies TNBC, Basal-Like and Claudin-Low breast cancer.** Cox proportional-hazards regression calculations were carried out in MedCalc based on ROC optimal cutoff percentiles, as indicated in the table. Tumor numbers in the high or low expression groups are indicated on the right. Analysis was carried out on the UNC cohort of breast tumors, subdivided as indicated in column two. CI = confidence interval, HR = hazards ratio. Tumor numbers (tumor #) in the high or low expression groups are indicated on the right.

Supplementary Table 3: Validation for the stratification of DMFS for Basal-like tumors by PMCA genes.

Covariate	Cox Proportial-Hazards Regression					ROC Optimal Cutoff	Tumor #	
	P-Value	HR	95% CI				Low	High
PMCA1 (high vs low)	0.0742	0.515	0.249	-	1.063	≥35.3% = High	32	60
PMCA2 (high vs low)	**0.0045**	0.216	0.076	-	0.618	≥62.5% = High	57	35
PMCA4 (high vs low)	0.1425	1.960	0.801	-	4.785	≥32.1% = High	29	63

Supplementary table 3: Validation for the stratification of DMFS for Basal-like tumors by PMCA genes. Cox proportional-hazards regression calculations were carried out in MedCalc based on ROC optimal cutoff percentiles, as indicated in the table. Tumor numbers in the high or low expression groups are indicated on the right. Validation analysis was carried out in the VDX [54] cohort. CI = confidence interval, HR = hazards ratio. Tumor numbers (tumor #) in the high or low expression groups are indicated on the right.

Supplementary Table 4: Secondary validation for the stratification of RFS for Basal-Like tumors by PMCA genes.

Gene	Cohort	Treatment	Logrank			Tumor #	
			**HR**	**95% CI**	**P-Value**	**Low**	**High**
PMCA1	**Basal-Like**	-	1.16	(0.89 - 1.50)	0.27	302	278
PMCA2			0.65	(0.50 - 0.85)	**0.0013**	245	335
PMCA4			1.24	(0.93 - 1.65)	0.14	196	384
PMCA1		CT	1.58	(0.83 - 3.00)	0.16	90	35
PMCA2			0.52	(0.28 - 0.99)	**0.043**	62	63
PMCA4			3.68	(1.44 - 9.39)	**0.0035**	39	86

Supplementary table 4: Secondary validation for the stratification of RFS for Basal-Like tumors by PMCA genes. Kaplan-Meier Log-rank curve analysis in all Basal-Like tumors or those receiving chemotherapy (CT) only based on relapse-free survival (RFS). The auto-select feature was used when assigning groups of high and low expression. CI = confidence interval, HR = hazards ratio. Tumor numbers (tumor #) in the high or low expression groups are indicated on the right. Secondary validation was carried out using the online tool, Kaplan-Meier Plotter [55].

A

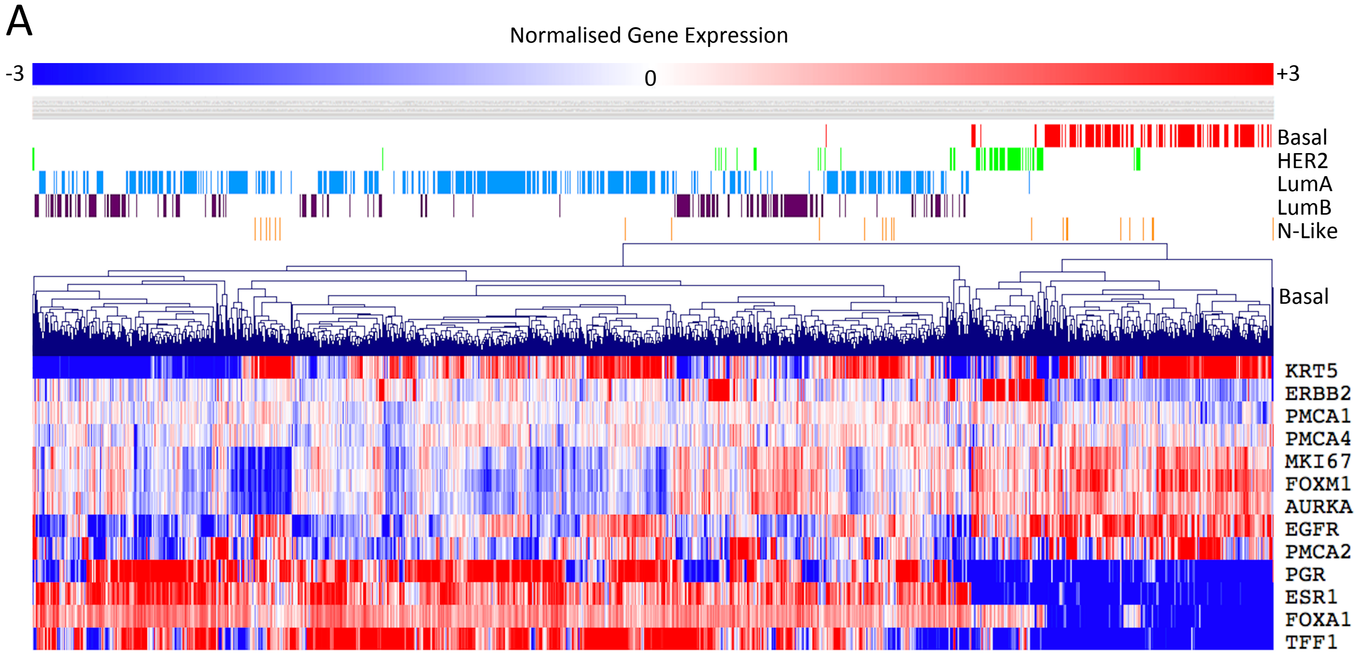

B

Gene	Correl. to PMCA2 (All)	P-Value	Correl. to PMCA2 (Basal)	P-Value
KRT5	0.232	6.9E-18	0.145	6.8E-02
ERBB2	-0.097	7.2E-04	0.030	7.2E-01
PMCA1	-0.045	1.3E-01	-0.228	2.7E-03
PMCA4	0.181	6.0E-11	0.084	3.1E-01
MKI67	0.067	2.2E-02	-0.012	8.9E-01
FOXM1	0.098	6.5E-04	-0.031	7.1E-01
AURKA	0.036	2.2E-01	-0.050	5.5E-01
EGFR	0.262	6.2E-23	-0.220	4.1E-03
PMCA2	-	-	-	-
PGR	-0.161	7.8E-09	0.135	9.2E-02
ESR1	-0.220	4.5E-16	0.135	9.2E-02
FOXA1	-0.300	1.2E-30	-0.115	1.5E-01
TFF1	-0.205	6.0E-14	-0.115	1.5E-01

Supplementary figure 1: PMCA2 is differentially expressed in breast cancer. **(A)**, normalised gene expression for PMCA genes and molecular markers of breast cancer. Tumor assignments to the intrinsic molecular subtypes (PAM50) are indicated above the heatmap. RNA-Seq data is sourced from TCGA and represents log-2 transformed mean-centred read counts (RSEM produce by TCGA). **(B)**, Pearson's correlation coefficients for genes in A compared to PMCA2 in all tumors and specifically the Basal-like breast tumours. P-values for correlations are listed.

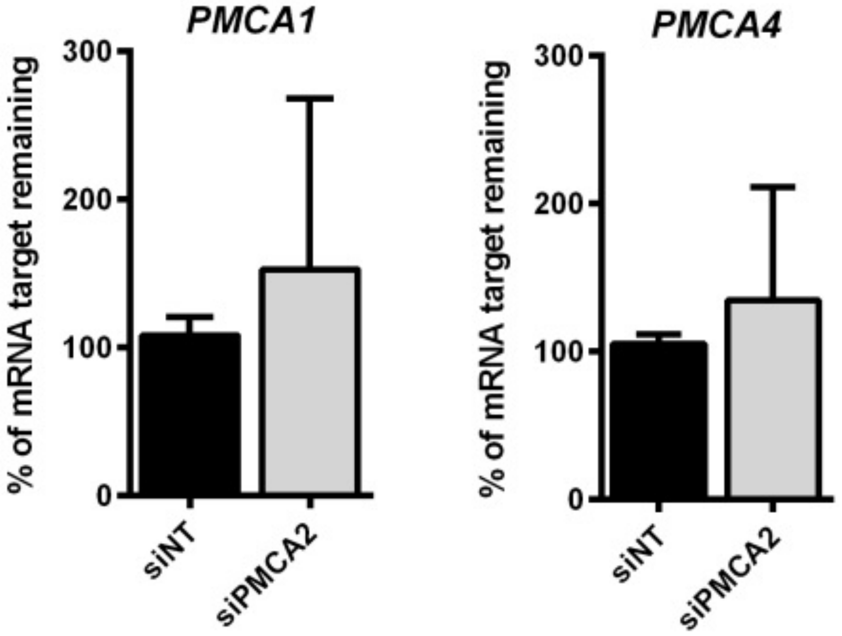

Supplementary figure 2: *PMCA* mRNA levels in MDA-MB-231 breast cancer cells with PMCA2 silencing. *PMCA1* and *PMCA4* mRNA levels, 120 h after transfection with non-targeting siRNA (siNT) or PMCA2 siRNA (siPMCA2). The data are mean ± SD (n = 3) and are from three independent experiments. P > 0.05, unpaired t-test.
